# The Stability and Evolution of Genes and Genomes

**DOI:** 10.3390/genes14091747

**Published:** 2023-08-31

**Authors:** Luigi Viggiano, René Massimiliano Marsano

**Affiliations:** Dipartimento di Bioscienze, Biotecnologie e Ambiente, Università degli Studi di Bari “Aldo Moro”, 70121 Bari, Italy

The existence of current species can be attributed to a dynamic interplay between evolutionary forces and the maintenance of genetic information. Genes and genomes are constantly evolving entities, shaped by a multitude of forces that maintain their stability while allowing for necessary changes. These forces include (but are not limited to) mutations, transposition, and natural selection.

Together, these forces ensure a delicate balance between preserving genetic information and generating crucial variability for species survival.

While mutations are the primary drivers of evolution, there are cellular mechanisms that counterbalance excessive variation and contribute to the stability of genes and genomes and preserve the faithful pass down of the genetic material from generation to generation.

In this Special Issue of *Genes*, titled “The Stability and Evolution of Genes and Genomes”, we have collected eight original research papers and four reviews. These contributions aim to explore various fields, such as the role of transposable elements, the epigenetic effect of heterochromatin on gene expression, karyotype variability, evolution of complex behavioral traits, the stabilization of genetic information in organelles, and viral genome evolution.

Determining what primordial forms of life looked like is a debated topic for which several theories have been proposed [1]. Among the unanswered questions is whether primordial bacteria had a single or a double envelope membrane. In their article, Léonard et al. [2] addressed the significant question of the identity of the last bacterial common ancestor, advancing the suggestive hypothesis that bacteria might have evolved from a common ancestor with a monoderm cell wall architecture. They suggested that the appearance of the outer membrane was not a unique event in evolution and that selective forces have led to the repeated adoption of such an architecture.

Viruses and bacteriophages are also among the earliest form of life [3]. Due to their fast replication, they are considered as exceptional models to study the evolution and the stabilization of genetic information. In particular, understanding how viruses generate new genetic information during evolution and how this information is stabilized and modified in a limited genomic size is currently a debated subject and a relevant issue in public health. In a highly interesting review [4], Pavesi discussed the origin, evolution, and adaptive conflict of overlapping genes, and the critical role of genes in the evolution of viral pathogenicity.

Karyoptype has long been used as a representative taxonomic character, although the karyotypes of closely related species often differ [5]. To resolve the questionable taxonomic structure of the Calomyscus genus [6], Romanenko et al. [7] analyzed karyotype plasticity in 14 specimens of the mouse-like hamsters collected in various Iranian locations through comparative cytogenetics approaches. In this paper, the authors provided a detailed description of the karyotype and concluded that it cannot be used as an unambiguous indicator of Calumyscus species rank. This paper confirms the entangled relationship between the evolutionary histories of living organisms and their phenotypic outcomes.

The evolution of genes can also impact sexual behavior and thus reproduction [8]. These are complex phenotypic traits that require combined methodological approaches to be dissected. Through a combination of transcriptomics, real time qPCR, and phylogenetics, Nieberding and collaborators provided a large-scale investigation of the genetic pathways underlying sex pheromone communication in the butterfly *Bicyclus anynana* [9]. Furthermore, in their review, Nieberding and collaborators [10] provide an interesting link between the genetic basis of behavioral variation and the evolution of social learning oviposition-related behavior. This paper could initially sound off topic; however, we have decided to include it because of its relevance in conjoining behavioral genetics and the evolution of complex traits.

Studying the relationship between gene evolution and environmental adaptation is crucial to understand how extant living organisms have originated. In a comparative study, Zhou et al. analyzed the amino acid substitution rate and natural selection of the OXPHOS genes in fig wasps [11] under the hypothesis that these genes have experienced adaptation to the compact, hypo-oxygenated, and dark environment of the syconia.

The regulation of the activity of transposable elements determines to what extent they can act as natural mutagens [12]. Two papers [13,14] showed that a *Drosophila melanogaster* ribosomal protein is able to bind TEs through its special histone-like domains and advanced the intriguing hypothesis that this interaction could reflect a regulation of the activity of transposable elements, especially in the heterochromatin.

Heterochromatin is a major structural feature of the eukaryotic genome stability, a specialized type of chromatin that contains a complex and still poorly understood genomic compartment, extremely enriched in repeats and transposable elements [15] in which expressed genes are rare but not completely absent [16,17]. Heterochromatin is also a hallmark of telomeres and centromeres, two important loci that stabilize chromosomes and ensure proper segregation. The effect of heterochromatic domains on gene expression is still poorly understood. *D. melanogaster* offers a precious model system for studying how stable and compact heterochromatic blocks influence resident genes and artificially inserted euchromatic coding sequences. Messina et al. [18] investigated on the epigenetic silencing of P-element reporter genes induced by transcriptionally active domains of constitutive heterochromatin in Drosophila melanogaster. The description of such a paradoxical phenomenon further entangles our current knowledge on the intricate mechanisms at the basis of heterochromatin structure, function, and stabilization.

Telomeres are specialized structures of the eukaryotic chromosome that allow for the stabilization of terminal genetic information on the chromosome [19]. Chromosome-ends shortening is a natural phenomenon which occurs in differentiated cells and is at the basis of the senescence process [20]. The genetic destabilization induced by telomere shortening in specific tissues or cell types can be potentially used as a biomarker of certain diseases. Zimnitskaya et al. surveyed the scientific literature in the field and suggested that the telomere length in leucocytes can be a promising marker of coronary heart disease [21].

An important aspect in gene evolution is how genetic code is used. The codon usage bias reflects a species-specific use of the expressed tRNA set which impacts the translation of all mRNAs [22]. In his review, Matsuo makes the point on the codon usage bias of histone-coding genes in Drosophila species and proposes a model to explain the GC-richness at the third position of codons in these genes [23].

Eukaryotic cells are internally compartmentalized. Mitochondria are semiautonomous organelles, functionally and genetically inter-dependent from the nucleus [24]. This form of symbiosis results in the destabilization of the mitochondrial genome (mtDNA) when mutations hit a subset of nuclear genes. Gilea and collaborators have reviewed the current scientific literature on the use of the budding yeast *S. cerevisiae* in the study of the mutations of nuclear genes associated with mtDNA instability [25]. This review also highlights the importance of using model organisms as tools to study genome stability.

In conclusion, the papers collected in this Special Issue cover various aspects of how genetic information evolves and is stabilized and emphasize the importance of further studies that combine different methodological approaches in order to provide a complete picture of the dynamics underlying the complex process of genome stabilization and its evolution.
